# Upfront Enzyme Replacement via Erythrocyte Transfusions for PNP Deficiency

**DOI:** 10.1007/s10875-021-01003-9

**Published:** 2021-02-27

**Authors:** Anna Eichinger, Horst von Bernuth, Cinzia Dedieu, Sebastian A. Schroeder, Giancarlo la Marca, Michael H. Albert, Fabian Hauck

**Affiliations:** 1 Department of Pediatrics, Dr. von Hauner Children’s Hospital, University Hospital, Ludwig-Maximilians-Universität München, Munich, Germany; 2 Department of Pediatric Pneumology, Immunology and Intensive Care, Charité - Universitätsmedizin Berlin, Berlin, Germany; 3 Berlin-Brandenburg Center for Regenerative Therapies, Berlin, Germany; 4 Department of Immunology, Labor Berlin GmbH, Berlin, Germany; 5 Department of Pediatric Hematology and Oncology, Charité - Universitätsmedizin Berlin, Berlin, Germany; 6 Newborn Screening, Biochemistry and Pharmacology Laboratory, Clinic of Pediatric Neurology, Meyer University Children’s Hospital, Florence, Italy; 7 Department of Experimental and Clinical Biomedical Sciences, University of Florence, Florence, Italy; 8 German Centre for Infection Research (DZIF), Munich, Germany; 9 Munich Centre for Rare Diseases (MZSE), Munich, Germany

To the Editor,

Purine nucleoside phosphorylase (PNP) deficiency is a rare metabolic disorder leading to combined immunodeficiency (CID) and neurological deficits such as developmental delay, ataxia, and motor impairment. PNP is highly expressed in red blood cells (RBC) and phosphorolyzes (deoxy)-inosine and (deoxy)-guanosine to (deoxy)-guanine and hypoxanthine. Due to the enzymatic block, PNP-deficient patients display elevated concentrations of purine (deoxy-)nucleosides which have lympho- and neurotoxic effects. For example, dGTP which accumulates in mitochondria inhibits the synthesis of mitochondrial DNA leading to apoptosis and contributes to the neurological deficits [1]. The metabolic block caused by PNP deficiency prevents uric acid formation and frequently results in hypouricemia [2]. PNP deficiency can be treated with HSCT leading to correction of CID, but neurological deficits often remain or progress. A pioneering proof-of-concept work in the 1980s has shown that erythrocyte transfusions improve metabolic and immunologic functions [3, 4]. With the implementation of TREC-based newborn screening and the possibility to screen for PNP deficiency by tandem mass spectrometry, which would be more sensitive, there is a growing need for managing asymptomatic newborn PNP patients (P), not only curing CID but also preventing neurological deficits [5].

We report a series of three neonates/infants who had received a genetic diagnosis of PNP deficiency prenatally (P1 and P2, siblings) or early in life (P3) because of familial index cases (Tab.S1). They all had sequential therapy with immediate and ongoing erythrocyte transfusions (ET) followed by HSCT (Fig. 1). Before the initial erythrocyte exchange transfusions (EET), PNP RBC enzyme activity was decreased to 4.8 ± 3.3 μmol/min/g hemoglobin (reference 60–100) and dried blood spot purine metabolites were elevated (e.g., guanosine 12.3 ± 5.5 μmol/l; reference <1.1, Fig. 1, Tab.S3–S5). Uric acid was only reduced in P3 (0.2 mg/dl uric acid on day 6 of life), while it was normal to slightly elevated in P1 and P2 (Fig.S2). While postnatal clinical examination and cerebral ultrasound (patients 1 and 2) showed no neurological abnormalities, there were varying degrees of immune cell reduction (Tab.S2). Initial EET was performed immediately after birth in P1 and P2, and on day 23 of life in P3. These erythrocyte exchange transfusions on day 1 of life led to an increase in PNP enzyme activity to 50 (P1) and 21 (P2) μmol/min/g hemoglobin (at d2 of life) and a normalization of purine metabolites (as exemplified by guanosine 0.5 ± 0.1 μmol/l) (Fig. 1, Tab.S4–S6). Thereafter, P1–P3 received regular ET/EET following individualized schemes that were guided by purine metabolites (Fig. 1, Tab.S4–S6). P1 received five additional ET every 11 ± 10 days with a volume of 15 ± 9 ml/kg without prior phlebotomy due to anemia caused by concomitant sickle cell disease (Fig. 1a and Tab.S4). P2 received nine additional EET every 12 ± 5 days with a volume of 14 ± 2 ml/kg preceded by 6 phlebotomies of 10 ± 4 ml/kg (Fig. 1b and Tab.S5). P3 received 2 additional ET every 24 ± 6 days with a volume of 24 ml/kg (day 43) and an unclear ET volume (day 71) (Fig. 1c and Tab.S6). After the initial EET, P1 and P2 had thrombocytopenia (41G/l and 28G/l) without bleeding signs and both received thrombocyte transfusions once. P1 had transient partial respiratory insufficiency with oxygen dependency via nasal cannula and P2 had mild fluid overload and received two doses of furosemide. P3 had mild thrombocytopenia after the first (108G/l) and after the second ET (138G/l). No further adverse events related to EET/ET were observed.

**Fig. 1:**
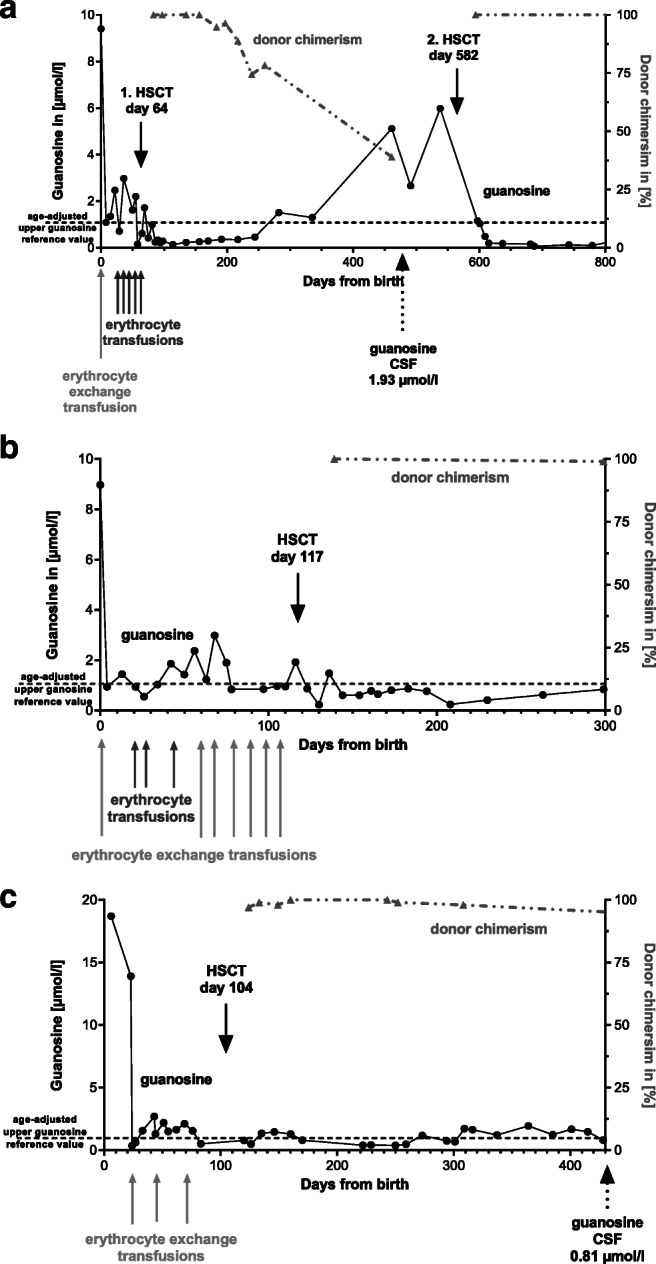
Clinical course of patients 1 (**a**), 2 (**b**), and 3 (**c**) indicating the time points of erythrocyte transfusions (EET/ETs) and HSCT, corresponding guanosine levels from dried blood spots (black, dots), and whole-blood chimerism after HSCT (gray, dashed-dotted line, triangles). Only ETs prior to HSCT, not those during aplasia after chemotherapy, are displayed. The upper reference value for guanosine (1.1 μmol/l) is marked with a black dashed line. CSF, cerebrospinal fluid

To achieve definitive cure of PNP deficiency, P1–P3 underwent allogeneic HSCT after busulfan/fludarabine/alemtuzumab-based myeloablative conditioning from mismatched family or matched unrelated donors at the age of 64 to 117 days, respectively (Tab.S1). HSCT of P1 was complicated by veno-occlusive disease that responded to defibrotide and by secondary graft failure (Fig. 1, Tab.S1 and S4). While peripheral blood donor chimerism was declining (72% on day +196), toxic purine metabolites increased in peripheral blood and cerebrospinal fluid. At this time point, P1 had no signs of CID, but neurological development slowed down. No ETs were performed between the diagnosis of secondary graft failure and second HSCT. Patient 1 was uneventfully re-transplanted at the age of 19 months from the same donor with an alternative myeloablative regimen and stem cell source (Tab.S1). P2 experienced acute graft-versus-host disease (GVHD) °III that responded to immunosuppressive therapy. The transplantation of P3 was uneventful without major complications. With a follow-up of 42, 32, and 37 months, respectively, all patients are alive and well with complete immune reconstitution and no chronic GVHD.

P1–P3 have successively reached developmental milestones at the follow-up visits; however, they have global developmental delay of varying degrees (Tab. S3). P1 and P3 were able to partially catch-up cognitive development while P2 has shown slower progress. Analyzing guanosine levels over time (Fig. 1), it is hard to determine different AUCs. Moreover, a variety of additional factors—e.g., different genotypes (P1/2 versus P3), intrauterine exposure to toxic purine metabolites [6], 2nd HSCT in P1, prolonged glucocorticoid use, and failure to thrive due to acute GVHD in P2—distinguish P1–P3 and may influence neurological development. Especially, the timing of exposure to substantially elevated neurotoxic metabolites might influence motor development. P3 who started ET/EETs at the age of 23 days and thus spent most of her newborn period with highly toxic levels cannot walk independently at the age of 3 years, while P1 and P2 are mobile on their own. Motor development, however, is most impaired in P1–P3 and P3 is doing better in other areas of the testing as compared to P1 and P2. Neuroimaging of P3 reveals global brain atrophy (Fig. S1) at the age of 28 months while there is none for P1 and P2.

Observations in single PNP patients suggest that neurological deficits can improve after early HSCT but can also progress especially after late HSCT [7, 8]. Clearly, larger HSCT series with long-term outcome data are lacking. Moreover, in the preexisting literature, no clear genotype-phenotype correlations were shown for the neurological outcome and the development of the children is also influenced by the acquired infections [9]. Thus, it is impossible to gauge the neurological outcome in a single patient and it is difficult to evaluate the effect of therapeutic interventions. Untreated murine PNP deficiency causes comparable neurological deficits that can be prevented by ERT [10]. Transient treatment with ET to provide PNP enzyme has established the proof of principle that this can lead to short-lasting biochemical and immunological improvement^34^. We here demonstrate in a case series of neonates/infants with PNP deficiency that this approach is safe as an upfront strategy followed by early definitive HSCT in pre-symptomatic patients. Initial EET should be performed under tight supervision of an experienced team in a distinguished expert center once the newborn is stable, and special attention should be paid to hypocalcemia, hypomagnesemia, and thrombocytopenia [11, 12]. Detection of purine metabolites with tandem mass spectrometry allows to guide ET regimens and to optimize the amount of red blood cell transfusions, thereby maintaining purine metabolites in the lower range and avoiding unnecessary transfusion-induced iron overload. The uric acid levels in our three patients were not corresponding well enough to purine metabolite levels and could not be used for transfusion guidance (Fig.S2), therefore justifying the more elaborate application of tandem mass spectrometry to directly measure purine metabolites. We show that this approach results in correction of purine metabolites and stabilizes the immunological phenotypes (Tab.S7–S8). We hypothesize that improved neuroprotection could be reached by immediate (EET), consecutive ET and long-lasting cross-detoxification (HSCT), even though significant long-term neurological benefit remains to be determined. We propose that the possible neurological benefit outweighs the possible complications of EET/ET that could be applied as a bridge to HSCT at the earliest possible time point. We envision that intrauterine ETs in PNP-deficient fetuses might further improve their neurocognitive development.

In non-malignant immunological and metabolic disorders, the degree of donor chimerism required for cure varies considerably across diseases and the particular cell lineages [13]. In PNP deficiency, the central nervous system requirement for PNP enzyme activity can only be targeted indirectly by HSCT. Thus, the conditioning regimen should allow for donor monocyte migration to the central nervous system and ensuing trans-differentiation into microglia-like cells. In animal models, this could best be achieved by busulfan-containing conditioning which we chose for P1–P3. Consequently, high donor myeloid chimerism should be obtained and properly monitored. While RBC represent the main source of PNP, their chimerism cannot be assessed easily in peripheral blood with standard genetic techniques. We show that monitoring purine metabolites can detect rising metabolites as a consequence of incomplete RBC and myeloid chimerism and propose it as a technique to monitor ADA and PNP deficiency after HSCT and gene therapy.

In summary, therapy of PNP deficiency with upfront enzyme replacement via EET/ET combined with definitive HSCT leads to immediate and permanent correction of metabolic and immunological phenotypes, while long-term neurological benefit remains to be determined. Monitoring of purine metabolites by tandem mass spectrometry is feasible for governing individual ET regimens and for detecting early graft failure especially of the erythroid lineage.

## Supplementary Information

ESM 1 (DOCX 112 kb)

ESM 2 (PDF 190 kb)

## Data Availability

Additional data is available in the supplementary material.
