# Supplementary material for: Upfront Enzyme Replacement via Erythrocyte Transfusions for PNP Deficiency
Source: J Clin Immunol. 2021 Feb 27;41(5):1112–5. doi: 10.1007/s10875-021-01003-9 (PMC8249256; doi:10.1007/s10875-021-01003-9)
Supplement: Supplementary file 1 — (DOCX 112 kb) [file 10875_2021_1003_MOESM1_ESM.docx]

**Supplementary Figure Legend**

**Figure S1.** Brain MRI of patient 3 at the age of 28 months. The MRI shows infra- and supratentorial cerebral atrophy with enlarged inner and outer brain cerebrospinal fluid spaces and normal age-related myelin maturation. A) T2w-weighed MRI scan, B) FLAIR MRI scan.

**Fig S2.** Clinical course of patients 1 (A), 2 (B) and 3 (C) indicating the time points of erythrocyte transfusions (EET/ETs) and HSCT, corresponding guanosine levels from dried blood spots (black, dots) and uric acid (grey, dashed line, triangles). Only ET prior to HSCT, not those during aplasia after chemotherapy are displayed. The upper reference value for guanosine (1.1 µmol/l) is marked with a black dashed line; the reference range of uric acid (1.2 – 6.2 µmol/l) is given with grey dashed lines.

**Supplementary Table**

Table S1. HSCT characteristics of patients 1-3. HSCT, including the tapering of immunosuppression, was performed according to institutional standards. CsA = ciclosporine A, MMF = mycophenolate mofetil, MMFD = mismatched familiar donor, MUD = matched unrelated donor.

|  | **Patient 1^a^** | | **Patient 2 ^a^** | **Patient 3** |
| --- | --- | --- | --- | --- |
| ***PNP* variant** | c.286-18G>A^b^ | | c.286-18G>A^b^ | c.244C>T^c^ |
|  | **1^st^ HSCT** | **2^nd^ HSCT** |  |  |
| **Age at HSCT (days)** | 64 | 582 | 117 | 104 |
| **Stem cell source and donor** | Bone marrow, MMFD (7/10) | PBSC, TCRα/β-, CD19-depleted, MMFD (7/10) | Bone marrow, MUD (9/10) | Bone marrow, MUD (10/10) |
| **Conditioning** | Alemtuzumab (0.4 mg/kg)  Fludarabine (5 mg/kg)  Busulfan (AUC 54,696 ng x h/ml)  Cyclophosphamide (20 mg/kg) | ATG Grafalon® (20 mg/kg)  Fludarabine (150 mg/m^2^)  Treosulfan (42 mg/m^2^)  Thiotepa (10 mg/kg)  Rituximab (200 mg/ m^2^) | Alemtuzumab (0.6 mg/kg)  Fludarabine (5.3 mg/kg)  Busulfan (AUC 78,760 ng x h/ml) | Alemtuzumab (0.5 mg/ kg)  Fludarabine (7.2 mg/kg)  Busulfan (AUC 79,172 ng x h/ml) |
| **Engraftment**  **Leukocytes** (> 1,000/µl)  **Neutrophils** (> 500/µl  **Thrombocytes** (> 50,000/µl) | day +6  day +14  day +27 | day +11  day +14  day +9 | day +16  day +16  day +19 | day +8  day +17  day +24 |
| **GvHD Prophylaxis** | MMF (until day +35)  Cyclophosphamide (day +3, day +4)  Tacrolimus (until day +121) | CsA (until day +30) | MMF (until day +28)  CsA (until day +233) | MMF (until day +110)  CsA (until day +273) |
| **GvHD Treatment** | none | | Prolonged CsA  Methylprednisolone  Cow-milk free nutrition | none |
| **Complications** | VOD, resolved  Autologous reconstitution | none | Acute GVHD °III (skin and gut), resolved | none |
| **Whole blood donor chimerism** | 100 % (day +720 after 2^nd^ HSCT) | | 95 – 99 % (day +364) | 99% (day +252)  89% (day +701) |

^a)^ Patient 1 and Patient 2 are sisters. ^b)^ PMID: 24767876. ^c)^ PMID: 22664165

Table S2. Immunological phenotype of patients 1-3 measured before first erythrocyte transfusion with cell amounts in [#/µl], immunoglobulin concentrations in [g/l] and T-cell receptor excision circles in copy numbers. Abnormal results are given in bold letters.

|  | **Normal range** | **Patient 1 ^a^** | **Patient 2 ^a^** | **Patient 3 ^b^** |
| --- | --- | --- | --- | --- |
| **Lymphocytes** | 1,750 – 8,000/µl | **1,584** | **774** | 1,100 |
| **CD3^+^ T cells** | 600 – 5,000/µl | 1,299 | **511** | 800 |
| **CD4^+^ T cells** | 400 – 3,500/µl | 1,014 | **372** | **390** |
| **CD8^+^ T cells** | 200 – 1,900/µl | **190** | **93** | **140** |
| **CD19^+^ B cells** | 40 – 1,100/µl | **143** | **23** | **50** |
| **CD16^+^CD56^+^ NK cells** | 100 – 1,900/µl | 111 | 209 | 260 |
| **IgM** | < 0.83 g/l | < 0.03 | 0.05 | 0.33 |
| **IgG** | 2.30 – 14.00 g/l | 11.85 | 11.2 | 5.17 |
| **IgA** | < 1.45 g/l | < 0.04 | < 0.04 | 0.16 |
| **TRECs** | < 8 | **4** | ***low*** | **0** |

^a)^First day of life of patient 1 and 2. ^b)^ 7^th^ day of life of patient 3.

Table S3a-c. Cognitive and motor neurological development of patients1-3 over time. The development was assessed with Bayley Scales of Infant and Toddler Development (BSID) up to the age of 42 months. Patient 1 had an additional assessment at 61 months using the Wechsler Preschool and Primary Scale of Intelligence, Fourth Edition (WPPSI-IV) and Movement-Assessment Battery for Children, Second Edition (M-ABC 2). The BSID-III score is scaled to a metric with a mean of 100 (standard deviation 15) and ranges from 40 to 160 (Value V). The result is also given in a percentile rank (PR) in [%] and its associated developmental age (DA) in months. The different test subscales are given separately. The BSID-II score is scaled to a metric with a mean of 100 (standard deviation 15). The results are summarized in the mental development index (MDI) and the psychomotor developmental index (PDI) with its associated developmental age. The WPPSI-IV is given as a composite score (mean of 100; standard deviation of 15) with percentile rank, 95% confidence interval and qualitative description. The M-ABC 2 gives a standard score for each domain (mean of 10, standard deviation of 3) and a percentile rank (mean of 50, standard deviation of 34). n.a. = not available.

*S3 a. Results of neurodevelopmental diagnostics of patient 1 at 30 months and at 5 years of age.*

| **Bayley Scales of Infant and Toddler Development (BSID-III)** | | | |
| --- | --- | --- | --- |
| **Numerical age at testing** | **30 months (11 months after 2^nd^ HSCT)** | | |
|  | **Value (V)** | **Percentile rank (PR)** | **Associated Developmental Age (DA)** |
| **Cognitive Development** | 55 | <1 | 21 |
| **Receptive Communication** | 63 | 1 | 22 |
| **Expressive Communication** |  |  | 29 |
| **Fine Motor Development** | 58 | 0.3 | 23 |
| **Gross Motor Development** |  |  | 16 |

| **Wechsler Preschool and Primary Scale of Intelligence, Fourth Edition (WPPSI-IV)** | | | | |
| --- | --- | --- | --- | --- |
| **Numerical age at testing** | **61 months (42 months after 2^nd^ HSCT)** | | | |
|  | **Composite Score** | **Percentile Rank** | **95% Confidence Interval** | **Qualitative Description** |
| **Verbal Comprehension** | 71 | 3 | 66 – 80 | Borderline |
| **Visual Spatial** | 75 | 5 | 69 – 87 | Borderline |
| **Fluid Reasoning** | 79 | 8 | 73 – 89 | Borderline |
| **Working Memory** | 84 | 14 | 77 – 94 | Low average |
| **Processing Speed** | 68 | 2 | 64 – 84 | Extremely low |
| **Full Scale IQ** | **72** | **3** | **67 – 80** | Borderline |

| **Movement Assessment Battery for Children (M-ABC 2)** | | |
| --- | --- | --- |
| **Numerical age at testing** | **61 months (42 months after 2^nd^ HSCT)** | |
|  | **Standard Score** | **Percentile Rank in [%]** |
| **Manual Dexterity** | 3 | 1 |
| **Ball Skills** | 6 | 9 |
| **Static and Dynamic Balance** | 4 | 2 |
| **Total Motor Score** | **2** | **0.4** |

*S3 b. Results BSID-III of patient 2 at 23 months and at 3 years of age. Due to the SARS-CoV-2 pandemic related restrictions, only a short evaluation was possible at the age of 36 months and a language scale could not be performed.*

| **Bayley Scales of Infant and Toddler Development (BSID-III)** | | | | | | | |  |
| --- | --- | --- | --- | --- | --- | --- | --- | --- |
| **Numerical age at testing** | | **23 months (19 months after HSCT)** | | | **36 months (32 months after HSCT)** | | |  |
|  | **V** | | **PR** | **DA** | **V** | **PR** | **DA** | |
| **Cognitive Development** | 70 | | 2 | 18 | n.a. | n.a. | 29 | |
| **Receptive Communication** | 91 | | 27 | 22 | n.a. | n.a. | n.a. | |
| **Expressive Communication** |  |  |  | 22 |  |  | n.a. | |
| **Fine Motor Development** | 61 | | 0.5 | 19 | n.a. | n.a. | 23 | |
| **Gross Motor Development** |  |  |  | 12 |  |  | 23 | |

*S3 c. Results BSID-II of patient 3 at 29 months and results BSID-III at 3 years of age. The psychomotor developmental index (PDI) is not available (n.a.); only the mental developmental index (MDI) is given. Patient 3 is – due to her ankle spasticity – not able to walk independently.*

| **Bayley Scales of Infant and Toddler Development (BSID-II)** | | | | | |  |
| --- | --- | --- | --- | --- | --- | --- |
| **Numerical age at testing** | **29 months (25 months after HSCT)** | | **41 months (37 months after HSCT)** | | |  |
|  | **V** | **DA** | **V** | **Confidence Interval (90%)** | **DA** | |
| **MDI** | 51 | 19 | 80 | 75 – 92 | 32 | |
| **PDI** | n.a. | n.a. | n.a. | n.a. | n.a. | |

Table S4. Purine metabolites of patient 1 over time. Values outside of the normal range are written in bold letters. The red lines indicate the time points of erythrocyte transfusions, including the ones necessary during allogeneic HSCT. The blue lines indicate the time points of the allogeneic HSCTs.

| **Days of life** | **Guanosine in [µmol/l]**  **(normal <1.1)** | **Inosine in [µmol/l]**  **(normal <16,8)** | **Deoxyguanosine in [µmol/l] (normal <0.1)** | **Deoxyinosine in [µmol/l] (normal <0.08)** |
| --- | --- | --- | --- | --- |
| 0 | **9,4** | **63,9** | **4,21** | **8,92** |
| 1 | 0,46 | 4,08 | 0 | 0 |
| 8 | 1,09 | 5,79 | 0 | 0 |
| 15 | **1,35** | 4,26 | 0,01 | **2,09** |
| 22 | **2,47** | 8,7 | 0,06 | **5,4** |
| 28 | **5,26** | 13,03 | 0,15 | **12,26** |
| 29 | 0,71 | 5,56 | 0,01 | **0,62** |
| 36 | **2,98** | **17,99** | 0,04 | **6,91** |
| 43 | **1,84** | 5,54 | 0,05 | **4,53** |
| 50 | **1,62** | 7,1 | 0,02 | **3,37** |
| 55 | **2,2** | 9 | 0,05 | **4,37** |
| 56 | 1,07 | 2,54 | 0,02 | **1,57** |
| 57 | 0,61 | 3,35 | 0,01 | **0,93** |
| 58 | 0,15 | 0,74 | 0,01 | **0,2** |
| 63 | 0,62 | 2,74 | 0,02 | **1,05** |
| 64 | **2,06** | 7,04 | 0,05 | **3,18** |
| 65 | 0,61 | 2,85 | 0 | **2,2** |
| 67 | 0,43 | 2,28 | 0,21 | **2,31** |
| 69 | **1,72** | 8,94 | **1,2** | **7,52** |
| 71 | 0,97 | 4,91 | 0 | 0 |
| 73 | 0,71 | 2,96 | 0 | 0 |
| 75 | 0,42 | 1,45 | 0 | 0 |
| 77 | 0,24 | 0,57 | 0 | 0 |
| 79 | 1,02 | 6,15 | 0 | 0 |
| 81 | 0,99 | 4,06 | 0 | 0 |
| 83 | 0 | 1,39 | 0 | 0 |
| 85 | **1,98** | 7,22 | **1,03** | 0 |
| 86 | 0,247 | 2,03 | 0,031 | 0 |
| 90 | 0,333 | 2,13 | 0,032 | 0 |
| 94 | 0,215 | 1,57 | 0,0212 | 0 |
| 98 | 0,287 | 2,51 | 0,025 | 0 |
| 95 | 0,561 | 6,89 | 0,0335 | 0 |
| 114 | 0,14 | 3,03 | 0 | 0 |
| 135 | 0,233 | 3,13 | 0 | 0 |
| 156 | 0,255 | 3,57 | 0 | 0 |
| 170 | 0,291 | 3,51 | 0 | 0 |
| 191 | 0,361 | 4,33 | 0 | 0 |
| 217 | 0,35 | 8,15 | 0,02 | **1,29** |
| 244 | 0,46 | 12,9 | 0,04 | **1,25** |
| 282 | **1,51** | **37,79** | **0,13** | **3,49** |
| 335 | **1,3** | **19,8** | **0,255** | **4,23** |
| 461 | **5,12** | **38,7** | **0,54** | **1,86** |
| 491 | **2,66** | 6,4 | **0,41** | **1,46** |
| 538 | **5,98** | **49** | **0,7** | **2** |
| 597 | **1,16** | 13,7 | 0 | 0 |
| 601 | 1,03 | 12,04 | 0 | 0 |
| 609 | 0,49 | 11,35 | 0 | 0 |
| 615 | 0,2 | 3,16 | 0 | 0,2 |
| 637 | 0,18 | 1,8 | 0 | 0 |
| 681 | 0,16 | 0,91 | 0 | 0 |
| 687 | 0,07 | 0,95 | 0 | 0 |
| 742 | 0,13 | 1,82 | 0 | 0 |
| 779 | 0,1 | 0,53 | 0 | 0 |
| 954 | 1,09 | 17,8 | 0 | 0,34 |
| 1715 | 1,29 | 21,3 | 0,03 | 0,65 |

Table S5. Purine metabolites of patient 2 over time. Values outside of the normal range are written in bold letters. The red lines indicate the time points of erythrocyte transfusions, including the ones necessary during allogeneic HSCT. The blue line indicates the time point of allogeneic HSCT.

| **Days of life** | **Guanosine in [µmol/l]**  **(normal <1.1)** | **Inosine in [µmol/l]**  **(normal <16,8)** | **Deoxyguanosine in [µmol/l] (normal <0.1)** | **Deoxyinosine in [µmol/l] (normal <0.08)** |
| --- | --- | --- | --- | --- |
| 0 | **8,97** | **44,72** | **6,75** | **20,71** |
| 1 | 0,65 | 2,78 | **0,14** | **1,02** |
| 13 | **1,44** | 3,84 | **0,16** | **0,56** |
| 21 | 0,94 | 1,94 | **0,22** | **0,78** |
| 26 | 0,55 | 1,94 | 0,06 | **0,21** |
| 34 | 1,03 | 5,58 | **0,15** | **1,05** |
| 42 | **1,86** | 5,96 | **0,68** | **2,89** |
| 50 | **1,43** | 4 | **0,5** | **1,99** |
| 56 | **2,37** | 4,89 | **1,45** | **5,77** |
| 63 | **1,23** | 10,58 | **0,34** | **9,06** |
| 68 | **2,98** | 13,18 | **0,71** | **8,27** |
| 75 | **1,9** | 8,28 | **0,3** | **4,46** |
| 78 | 0,84 | 2,7 | **0,16** | **1,54** |
| 97 | 0,85 | 6,66 | 0,06 | **0,62** |
| 105 | 0,97 | 3,12 | **0,25** | **1,31** |
| 110 | 0,95 | 5,52 | 0,09 | **0,58** |
| 116 | **1,92** | 8,29 | **0,55** | **2,59** |
| 123 | 0,87 | 2,69 | **0,19** | **0,63** |
| 130 | 0,23 | 0,87 | 0 | 0 |
| 136 | **1,48** | 5,95 | 0,06 | **0,22** |
| 144 | 0,61 | 5,9 | 0,03 | **0,38** |
| 154 | 0,61 | 5,19 | 0 | **0,31** |
| 161 | 0,78 | 6,69 | 0 | **0,35** |
| 165 | 0,65 | 6,16 | 0 | **0,51** |
| 173 | 0,8 | 12,44 | 0,05 | **0,44** |
| 183 | 0,87 | **21,56** | 0,04 | **0,59** |
| 194 | 0,77 | **17,65** | 0,04 | **0,41** |
| 208 | 0,24 | 0,6 | 0 | 0 |
| 230 | 0,41 | 8,06 | 0,03 | **0,2** |
| 263 | 0,62 | 10,7 | 0 | **0,27** |
| 299 | 0,84 | 13,23 | 0 | **0,44** |
| 320 | 0,23 | 4,88 | 0,01 | **0,43** |
| 362 | 0,82 | 13,29 | 0,03 | **0,49** |
| 390 | 0,76 | 13,6 | 0,03 | **0,77** |
| 950 | 0,3 | 2,21 | 0 | 0 |

Table S6. Purine metabolites of patient 3 over time. Values outside of the normal range are written in bold letters. The red lines indicate the time points of erythrocyte transfusions, not the ones including the ones necessary during allogeneic HSCT. The blue line indicates the time point of allogeneic HSCT.

| **Days of life** | **Guanosine in [µmol/l]**  **(normal <1.1)** | **Inosine in [µmol/l]**  **(normal <16,8)** | **Deoxyguanosine in [µmol/l] (normal <0.1)** | **Deoxyinosine in [µmol/l] (normal <0.08)** |
| --- | --- | --- | --- | --- |
| 6 | **18,7** | **77,7** | **6,9** | **6,9** |
| 23 | **13,9** | **64,7** | **3,9** | **6,6** |
| 24 | 0,36 | 1,6 | 0 | 0 |
| 27 | 0,61 | 3,24 | 0 | 0 |
| 33 | **1,56** | 5,3 | 0,08 | **0,33** |
| 43 | **2,7** | 8,3 | **0,28** | **1,9** |
| 44 | **1,3** | 3,7 | 0 | 0,08 |
| 51 | **2,2** | 8,9 | **0,18** | **1,47** |
| 55 | **1,5** | 4,83 | **0,3** | **1,93** |
| 62 | **1,64** | 9,22 | **0,91** | **5,31** |
| 69 | **2,12** | 7,02 | **1,29** | **7,82** |
| 76 | **1,55** | 7,93 | 0,07 | **0,59** |
| 83 | 0,51 | 3,53 | **0,12** | **1,84** |
| 120 | 0,78 | 3,43 | **0,24** | **1,54** |
| 126 | 0,49 | 3,74 | 0,03 | **0,33** |
| 135 | **1,35** | 12,7 | 0,05 | **0,71** |
| 146 | **1,46** | 16,3 | 0,07 | **1,02** |
| 160 | **1,3** | 10,7 | 0,07 | **0,68** |
| 170 | 0,8 | 8,7 | 0,03 | **0,19** |
| 222 | 0,4 | 1,46 | 0 | 0 |
| 229 | 0,42 | 3,66 | 0 | 0 |
| 250 | 0,38 | 2,59 | 0 | **0,23** |
| 259 | 0,48 | 2,65 | 0 | 0 |
| 273 | **1,18** | 12,07 | 0,05 | **0,48** |
| 294 | 0,74 | 7,37 | 0 | **0,38** |
| 301 | 0,68 | 6,83 | 0 | **0,47** |
| 309 | **1,72** | **18,71** | 0 | **0,9** |
| 316 | **1,64** | **19,16** | 0 | **0,83** |
| 337 | **1,22** | **21,02** | 0,04 | **0,74** |
| 364 | **1,94** | **20,84** | 0,08 | **0,88** |
| 385 | **1,24** | 13,75 | 0,05 | **0,65** |
| 401 | **1,68** | **21,83** | 0,06 | **0,7** |
| 415 | **1,47** | **18,26** | 0,03 | **0,58** |
| 428 | 0,81 | 8,88 | 0,02 | **0,34** |

Table S7. Immunological blood values (leukocytes, lymphocytes, IgG, CD3^+^ T cells, CD4^+^ T cells, CD8^+^ T cells, CD19^+^ B cells and CD56^+^ NK cells) of patient 1 over time up to the first allogeneic HSCT on day 64 of life (conditioning chemotherapy started on day 55 of life). Values outside of the normal range are written in bold letters. The red lines indicate the time points of erythrocyte transfusions. The blue lines indicate the time points of the two allogeneic HSCTs. After the HSCT the days (d+)/years (a+) after the corresponding HSCT are given in brackets. Additionally, patient 1 received pneumocystis prophylaxis from day 8 until day 53 of life, after the first (day +29 until day +281) and second (day +18 until day + 180) allogeneic HSCT. Intravenous immunoglobulins were given eleven times from day 6 to day 756 of life (day 6, 50, 54, 108, 135, 156, 183, 570, 587, 591, 756).

| **Days of life** ^a)^ | **Leukocytes**  **[/µl]** | **Lymphocytes**  **[/µl]** | **IgG**  **[mg/dl]** | **CD3^+^ T cells**  **[/µl]** | **CD4^+^ T cells**  **[/µl]** | **CD8^+^ T cells**  **[/µl]** | **CD19^+^ B cells**  **[/µl]** | **CD56^+^ NK cells**  **[/µl]** |
| --- | --- | --- | --- | --- | --- | --- | --- | --- |
| 0 | 8500 | 1960 | 1185 | 1299 | 1014 | 190 | 143 | 111 |
| 0 | 6100 | **770** |  |  |  |  |  |  |
| 1 | 6300 | **790** | 785 | **347** | **286** | **45** | **29** | **20** |
| 8 | 6000 | 2320 |  |  |  |  |  |  |
| 15 | 3600 | **1680** | 1063 | **1146** | **898** | **186** | **93** | **232** |
| 22 | **5400** | 2930 | 897 | **2070** | **1604** | **350** | **379** | **292** |
| 27 | **5000** | 2430 |  |  |  |  |  |  |
| 28 | **3300** | **1150** | 593 |  |  |  |  |  |
| 28 | **6900** | **1630** |  |  |  |  |  |  |
| 29 | **4100** | **1420** |  |  |  |  |  |  |
| 30 | **4300** | **1680** |  |  |  |  |  |  |
| 36 | **3200** | **1670** | 564 | **1165** | **948** | **150** | **399** | **67** |
| 43 | **2600** | **1310** | 484 | **884** | **715** | **117** | **299** | **78** |
| 50 | **2900** | **1510** | 404 |  |  |  |  |  |
| 55 | **2500** | **1500** | **892** | **991** | **808** | **122** | **488** | **46** |
| 56 | **1400** | **150** |  |  |  |  |  |  |
| 57 | **400** | **40** |  |  |  |  |  |  |
| 57 | **300** | **40** |  |  |  |  |  |  |
| 58 | **800** |  |  |  |  |  |  |  |
| 59 | **700** | **20** |  |  |  |  |  |  |
| 60 | **700** | **40** |  |  |  |  |  |  |
| 61 | **500** | **30** |  |  |  |  |  |  |
| 62 | **600** | **20** | **741** |  |  |  |  |  |
| 63 | **400** |  |  |  |  |  |  |  |
| 64 | **300** |  |  |  |  |  |  |  |
| 135  (d +71) | 6500 | **1200** | 580 | **210** | **148** | **37** | **568** | 383 |
| 156  (d +92) | 11200 | **950** | 697 | **302** | **212** | **30** | **444** | 202 |
| 461  (a +1) | 8100 | 3380 | **437** | 2381 | 1565 | 714 | **476** | 510 |
| 644  (d +62) | **3000** | **650** | **448** | **304** | **132** | **119** | **125** | 205 |
| 681  (d +99) | **2700** |  | 574 | **266** | **138** | **83** | **376** | 248 |
| 756  (d +174) | 7180 | 1940 | **378** | 1183 | 756 | **291** | 465 | 271 |
| 954  (a +1) | 8400 | 4630 | 518 | 2818 | 1525 | 924 | 832 | 924 |
| 1302  (a +2) | 9250 | 3280 | 787 | 2165 | 1365 | 633 | 533 | 599 |
| 1715  (a+3) | 6740 | 3650 | 866 | 2548 | 1383 | 801 | 582 | 473 |

^a)^ Age-adjusted normal values for the lymphocyte subsets can be found at PMID: 14610491.

Table S8. Immunological blood values (leukocytes, lymphocytes, IgG, CD3^+^ T cells, CD4^+^ T cells, CD8^+^ T cells, CD19^+^ B cells and CD56^+^ NK cells) of patient 2 over time up to the allogeneic HSCT on day 117 of life (conditioning chemotherapy started on day 110 of life). Values outside of the normal range are written in bold letters. The red lines indicate the time points of erythrocyte transfusions. The blue line indicates the time point of the two allogeneic HSCTs. After the HSCT the days (d+)/years (a+) after the corresponding HSCT are given in brackets. Additionally, patient 2 received pneumocystis prophylaxis from day 1 until day 108 of life and after the allogeneic HSCT (day +20 until day +702). Intravenous immunoglobulins were given 14 times from day 36 to day 427 of life (day 36, 50, 68, 78, 97, 109, 158, 215, 256, 284, 334, 362, 390, 427).

| **Days of life** ^a)^ | **Leukocytes**  **[/µl]** | **Lymphocytes**  **[/µl]** | **IgG**  **[g/l]** | **CD3^+^ T cells**  **[/µl]** | **CD4^+^ T cells**  **[/µl]** | **CD8^+^ T cells**  **[/µl]** | **CD19^+^ B cells**  **[/µl]** | **CD56^+^ NK cells**  **[/µl]** |
| --- | --- | --- | --- | --- | --- | --- | --- | --- |
| 0 | 9990 |  |  |  |  |  |  |  |
| 0 | 12900 | **1490** | 11,2 | 611 | **372** | **93** | **23** | 209 |
| 0 | **19500** | **840** |  |  |  |  |  |  |
| 0 | **7570** | **420** | 8,6 |  |  |  |  |  |
| 1 | 10100 | **800** |  |  |  |  |  |  |
| 1 | 9890 | **1120** |  | 679 | 501 | **145** | **32** | **81** |
| 6 | **7710** | 1980 | 10,2 |  |  |  |  |  |
| 13 | **5690** | 2030 |  |  |  |  |  |  |
| 14 | 8310 | 2550 |  |  |  |  |  |  |
| 21 | **4220** | **1820** | 6,39 | **990** | **775** | **158** | **86** | 301 |
| 25 | **4170** | **1750** | 5,89 |  |  |  |  |  |
| 26 | **5110** | **1770** | 5,07 | **1252** | **966** | **215** | **304** | **197** |
| 27 | **6640** | **1530** |  |  |  |  |  |  |
| 34 | **5920** | **2030** | 4,72 |  |  |  |  |  |
| 42 | **2950** | **1390** | 7,83 |  |  |  |  |  |
| 50 | **3280** | **1480** |  |  |  |  |  |  |
| 56 | **2830** | **1230** | 8,91 |  |  |  |  |  |
| 63 | **3110** | **1160** | 7,8 | **713** | **564** | **104** | **276** | 127 |
| 68 | **2690** | **980** | 7,24 |  |  |  |  |  |
| 75 | **2820** | **1130** | 8,9 |  |  |  |  |  |
| 78 | **2820** | **1170** | 8,41 |  |  |  |  |  |
| 88 | **2720** | **1010** | 9,67 |  |  |  |  |  |
| 95 | **2340** | **1090** | 8,4 |  |  |  |  |  |
| 97 | **3120** | **1300** | 7,01 |  |  |  |  |  |
| 105 | **2610** | **850** | 8,96 |  |  |  |  |  |
| 109 | **2610** | **910** | 8,43 |  |  |  |  |  |
| 110 | **2890** |  | 11 |  |  |  |  |  |
| 111 | 7310 |  |  |  |  |  |  |  |
| 112 | **2100** |  |  |  |  |  |  |  |
| 113 | **2880** |  |  |  |  |  |  |  |
| 114 | **1700** |  |  |  |  |  |  |  |
| 115 | **1170** |  |  |  |  |  |  |  |
| 116 | **540** |  | 8,05 |  |  |  |  |  |
| 117 | **450** | **30** |  |  |  |  |  |  |
| 215  (d +98) | 3850 | **380** | **1,94** | **216** | **181** | **27** | **119** | **46** |
| 299  (d +182) | 6890 | **1160** | 4,59 | **586** | **445** | **117** | **375** | **187** |
| 440  (d +323) | 10200 | 2850 | 7,12 | 1856 | 1257 | 486 | 628 | 343 |
| 481  (a +1) | 7980 | 2890 | 5,12 | 1896 | 1178 | 575 | **172** | 747 |
| 950  (a +2) | 7110 | 3830 | 6,83 | 3072 | 2035 | 768 | 653 | 115 |

^a)^ Age-adjusted normal values for the lymphocyte subsets can be found at PMID: 14610491.
