# Supplementary figures and images for: Upfront Enzyme Replacement via Erythrocyte Transfusions for PNP Deficiency
Source: J Clin Immunol. 2021 Feb 27;41(5):1112–5. doi: 10.1007/s10875-021-01003-9 (PMC8249256; doi:10.1007/s10875-021-01003-9)

Figure S1

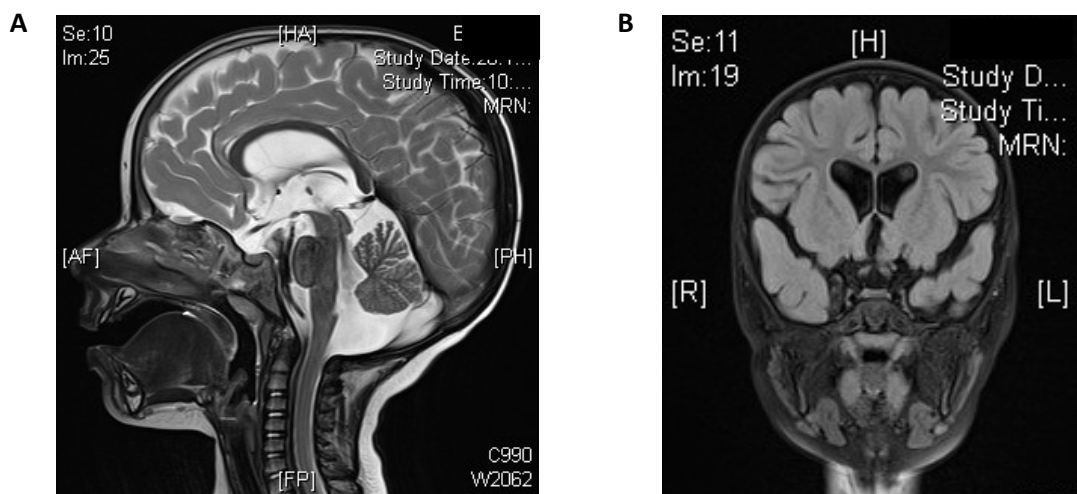

Figure S2    A

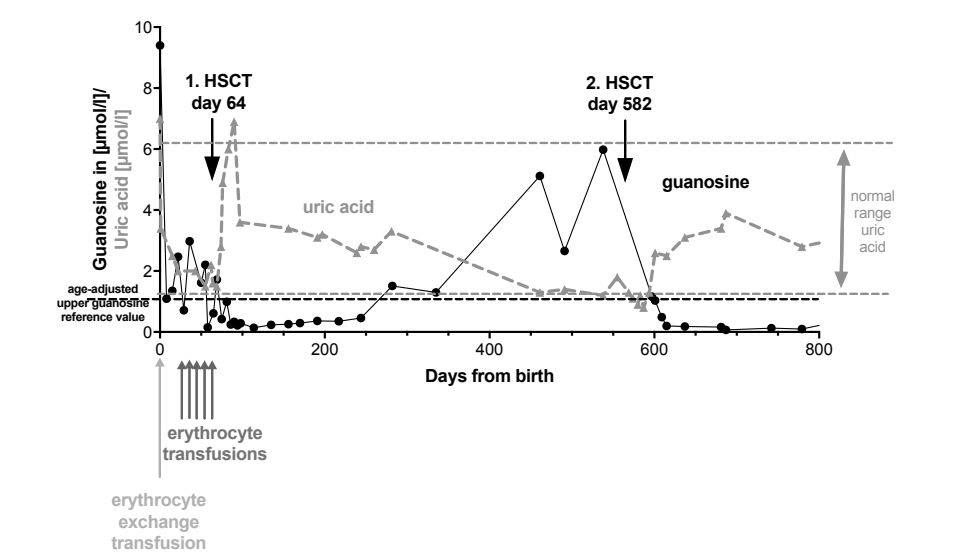

B

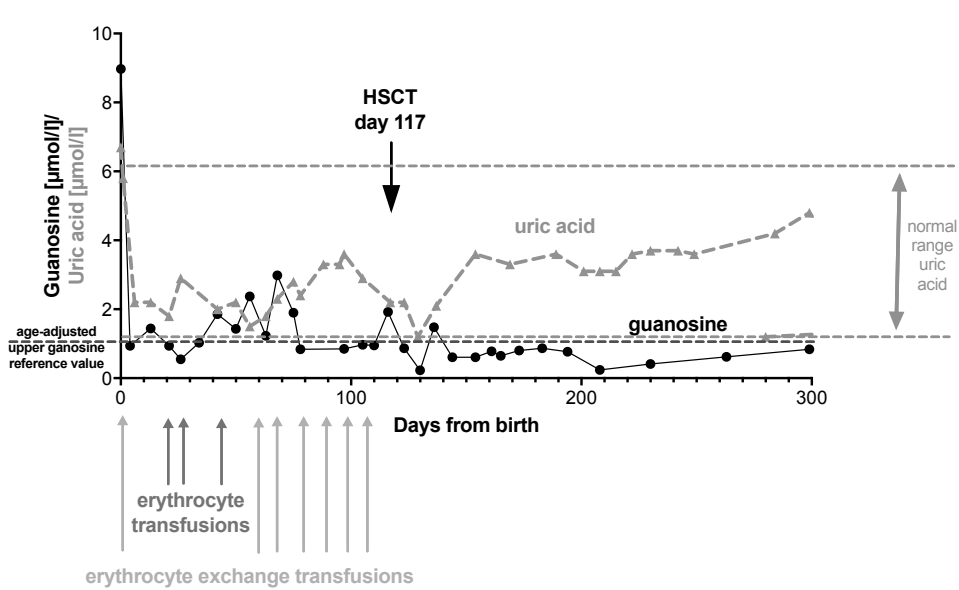

C

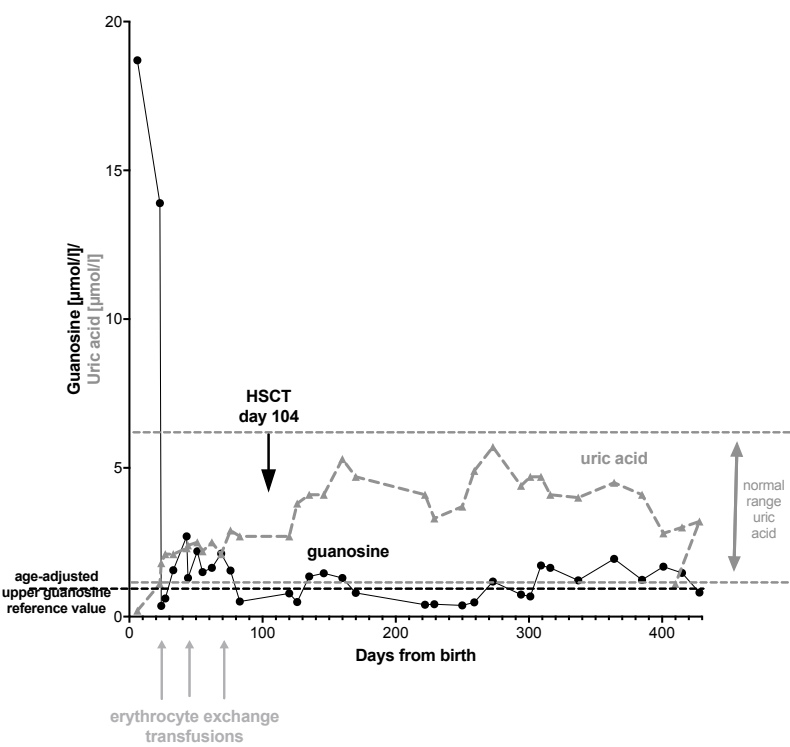

Supplement: Supplementary file 2 — (PDF 190 kb) [file 10875_2021_1003_MOESM2_ESM.pdf]
